# Hyponatremia Prevalence in Decompensated Chronic Liver Disease: Insights from a Tertiary Care Hospital

**DOI:** 10.7759/cureus.68907

**Published:** 2024-09-07

**Authors:** Muhammad Usman Azam, Najam-us-Sehar Saeed, Salman Javed, Muuhammad Yousuf Y Memon, Muhammad Asad Aftab, Muhammad Nabeel Shafqat, Hafiz Zeeshan Sadiq, Arman Maqbool, Fasih Mand Khan, Faizan Zahoor

**Affiliations:** 1 Department of Medicine, Gujranwala Medical College/Teaching Hospital, Gujranwala, PAK; 2 Department of Gastroenterology and Hepatology, Gujranwala Medical College/Teaching Hospital, Gujranwala, PAK; 3 Department of Gastroenterology and Hepatology, Services Institute of Medical Sciences, Lahore, PAK; 4 Department of Gastroenterology, King Saud Hospital, Unaizah, SAU; 5 Department of Medicine, St. Barnabas Hospital Health System, New York, USA; 6 Department of Medicine, Aziz Bhatti Shaheed Teaching Hospital, Gujrat, PAK; 7 Department of Medicine, FMH (Fatima Memorial Hospital) College of Medicine and Dentistry, Lahore, PAK; 8 Department of Medicine, DHQ (District Headquarter) Teaching Hospital, Gujranwala, PAK

**Keywords:** bmi, chronic liver disease, decompensated cirrhosis, encephalopathy, hyponatremia

## Abstract

Background: Liver cirrhosis is a prominent global contributor to mortality, and hyponatremia is a common complication in patients with decompensated chronic liver disease (DCLD). Hyponatremia is characterized by kidney impairment when eliminating solute-free water. The presence of contradictory findings in existing literature prompted this study.

Objective: The objective of this study was to determine the prevalence of hyponatremia in patients with DCLDs presenting at a tertiary care hospital.

Methodology: This six-month cross-sectional study was performed at the Allied Institute of Medical Sciences Teaching Hospital in Gujranwala, Pakistan, from January 2022 to June 2022. A total of 133 patients were selected as subjects. Researchers took blood samples from these patients and sent the samples to the hospital pathology lab for evaluation of serum sodium levels. If sodium levels were ≤130 mmol/L, the patient was considered to have hyponatremia. All information was recorded on proforma.

Results: The mean age of patients was 47.68 ± 12.89 years. Overall, 80 (60.15%) were male, and 53 (39.85%) female. The mean BMI of patients was 23.20 ± 3.11 kg/m^2^ and the average duration of DCLD was 7.24 ± 4.12 years. Among participants, 48 (36.09%) patients had hyponatremia, whereas 85 (63.91%) did not have hyponatremia. The mean sodium level was 132.39 ± 11.37 mEq/L. Stratified analysis based on patient age revealed that among patients aged 21-45 years, 27 (45.8%) had hyponatremia, whereas, in the group aged 46-70 years, 21 (28.4%) had hyponatremia with a p-value < 0.05. Stratification of the basis of BMI, among underweight patients, all eight (100%) had hyponatremia, whereas of overweight patients, 14 (31.1%) had hyponatremia. This difference was statistically significant (p < 0.05).

Conclusion: The prevalence of hyponatremia was notably elevated among individuals suffering from DCLD. Age and BMI were the most common risk factors for hyponatremia among subjects with DCLD. This study recommends that patients with DCLD should have their serum sodium levels screened at regular intervals to prevent complications, including encephalopathy, which occurs particularly in younger and underweight DCLD patients.

## Introduction

Cirrhosis is histologically defined as the formation of regenerative nodules encased by fibrous bands as a result of chronic liver inflammation [1]. Decompensated chronic liver disease (DCLD) refers to acute deterioration in liver function in patients with cirrhosis. DCLD can lead to a wide spectrum of complications, including but not limited to thrombocytopenia, coagulopathy, ascites, bleeding varices, hepatic encephalopathy, hepato-renal syndrome, hyponatremia, anasarca, and jaundice [2,3]. Among these, hyponatremia is a common and challenging complication that frequently coexists with end-stage liver disease. Hyponatremia is commonly diagnosed by the patient’s serum sodium level testing below 130 mEq/L, with values under 120 mEq/L considered a sign of severe hyponatremia [4,5]. As liver cirrhosis progresses, hyponatremia becomes more prevalent and severe [6]. Hyponatremia has also been linked to a 55% increase in the risk of death for patients with DCLD and places a major burden on health resources [7].

In patients with DCLD, the kidneys’ ability to remove free water is impaired, resulting in increased water retention relative to sodium retention, which in turn causes diminished serum sodium concentration and hypo-osmolality. Non-osmotic hypersecretion of arginine vasopressin from the neurohypophysis is another major cause of hyponatremia, which is complexly related to circulatory dysfunction [8,9].

Hyponatremia has been recognized as an indicator of advanced liver dysfunction, and it significantly increases patient morbidity and mortality [10-12]. Due to its significant prognostic value, hyponatremia diagnoses are included in the prognostic model for end-stage liver disease (MELD). Some research suggests the frequency of hyponatremia among DCLD patients is 70-73% [13,14]. However, a study based in the United States found hyponatremia in 21.6% of patients with cirrhosis and ascites [7]. In another study, the occurrence of dilutional hyponatremia, categorized by serum sodium levels of ≤135 mmol/L, ≤130 mmol/L, and ≤125 mmol/L, stood at 20.8%, 14.9%, and 12.2%, respectively [15].

Severe hyponatremia (<120 mEq/L) is associated with a high mortality rate, particularly in alcoholics, whose mortality rate with the condition is over 50% [16]. Similarly, cirrhotic patients awaiting transplant who have persistent ascites and low serum sodium levels have a high mortality risk despite low severity scores. The independent predictors, ascites and hyponatremia, are indicative of hemodynamic decompensation [17,18].

The literature suggests a strong correlation exists between DCLD and the risk of hyponatremia; however, the results have not been consistently supported by other studies. Moreover, no local studies have been conducted on this topic; local research is needed to investigate the severity of the issue among the local population. This study therefore aims to determine the prevalence of hyponatremia in patients with DCLD to better gather local evidence supporting the benefits of routine serum sodium level screenings in patients with DCLD.

## Materials and methods

Study sample and design

This six-month, cross-sectional research study was conducted at the Department of Gastroenterology at the Teaching Hospital of Gujranwala Medical College, Pakistan, between January 2022 and June 2022. The study was approved by the Institutional Review Board of Gujranwala Medical College/Teaching Hospital (approval number: 377/22).

Inclusion and exclusion criteria

All participants aged 18-70 years, regardless of gender, who presented with DCLD for regular medical assessment in the outpatient department of the hospital's gastroenterology unit were included in the study. Participants with comorbid conditions (i.e., renal problems; creatinine > 1.3 gm/dl), anemia (hemoglobin (Hb) < 10 g/dl), or thyroid problems (thyroid-stimulating hormone (TSH) > 5 IU/L, low T3, high T4 as per reference lab) were excluded. Participants who had taken saline solution for hyponatremia within the last 48 hours were also excluded.

Sample size

The sample size was 133 and the results were calculated with a 95% confidence level, a 7% margin of error, and an expected percentage of hyponatremia (i.e., 21.6% in DCLD patients). Samples were collected with a non-probability consecutive sampling technique.

Data collection

A total of 133 participants fulfilling the study criteria were selected from the outpatient department after obtaining informed consent. The demographic characteristics of the participants, including age, gender, BMI, and duration of DCLD were recorded. Blood samples were collected in 3 cc syringes, stored in vials, and sent to the hospital laboratory for measurement of serum sodium level screening. Patients were considered to have hyponatremia if serum sodium levels were below 130 mEq/L. All information was recorded in a form (see Appendices).

Data analysis

The data were loaded into IBM SPSS Statistics for Windows, Version 21.0 (Released 2012; IBM Corp., Armonk, New York, United States) for analysis. Means and standard deviations (SD) were calculated for quantitative parameters, which included age, BMI, and duration of DCLD. Gender and the presence of hyponatremia, classified as qualitative variables, were represented in terms of frequency and percentage. The dataset was stratified based on age, gender, BMI, and DCLD duration. To assess the variation of hyponatremia across these stratified groups, a chi-square test was implemented using a significance level of p ≤ 0.05.

## Results

A total of 133 patients were included in this study. The mean age (±SD) of the participants was 47.68±12.89 years; 80 (60.15%) were male and 53 (39.85%) female. The mean BMI (±SD) of patients was 23.20±3.11 kg/m^2^, and the mean duration of DCLD (±SD) was 7.24±4.1 years (Table 1).

**Table 1:** Demographic characteristics of study participants (N=133) DCLD: decompensated chronic liver disease

Characteristics	Epidemiological Distribution
Male, n (%)	80 (60.15%)
Female, n (%)	53 (39.85%)
Age (years), mean±SD	47.68±12.89
BMI (Kg/m^2^), mean±SD	23.20±3.11
Duration of DCLD (years), mean±SD	7.24±4.12

Overall, the mean sodium level (±SD) for all participants was 132.39±11.37 mEq/L. There were 48 (36.09%) patients with hyponatremia and 85 (63.91%) with normal sodium levels. Among patients with hyponatremia, the mean sodium level (±SD) was 118.13±5.22 mEq/L, whereas, in patients without hyponatremia, the mean sodium level was 140.28±3.11 mEq/L.

When data were stratified based on patient age, among patients aged 21-45 years, 27 (45.8%) were found to have hyponatremia, whereas, in the group aged 46-70 years, 21 (28.4%) had hyponatremia. This difference was found to be statistically significant (p < 0.05). In the stratified analysis based on participants' gender, among male patients, 28 (35.0%) had hyponatremia, whereas, in the female patient group, 20 (37.7%) had hyponatremia. However, this difference in gender groups was not statistically significant (Table 2).

**Table 2:** Stratified analysis of prevalence of hyponatremia according to age, gender, BMI, and duration of DCLD DCLD: decompensated chronic liver disease

Stratification Variables		Normal Sodium Levels	Hyponatremia	P-value
Age (years), n (%)	21–45	32 (54.2%)	27 (45.8%)	0.038
	46–70	53 (71.6%)	21 (28.4%)	
Gender, n (%)	Male	52 (65%)	28 (35.01%)	0.748
	Female	33 (62.3%)	20 (37.7%)	
BMI (kg/m^2^), n (%)	Underweight	0 (0.0%)	8 (100%)	0.001
	Normal	54 (67.5%)	26 (32.5%)	
	Overweight	31 (68.9%)	14 (31.1%)	
Duration of DCLD, n (%)	<10 years	60 (63.2%)	35 (36.8%)	0.051
	≥10 years	25 (65.8%)	13 (34.2%)	

In stratification analysis based on BMI it was seen that, among underweight patients, all eight (100%) had hyponatremia, whereas among participants with normal BMIs, 26 (32.5%) had hyponatremia. Of overweight patients, 14 (31.1%) had hyponatremia and the results were statistically significant (p < 0.05). When the data were stratified by duration of DCLD, of patients who had had DCLD for less than 10 years, 35 (36.8%) had hyponatremia, and, in those with DCLD for ≥ 10 years, only 13 (34.2%) had hyponatremia. In this case, the difference was statistically insignificant (p > 0.05) (Table 2).

## Discussion

A considerable number of patients with DCLD have abnormal serum sodium levels, with hyponatremia being the most common type. In our study, hyponatremia was also the most common finding, with no patients demonstrating serum sodium levels above 145 mEq/L. In this study, 48 individuals (36.09%) exhibited hyponatremia, and among those patients, the average sodium level was 118.13±5.22 mEq/L, whereas in participants without hyponatremia, the average sodium level was 140.28±3.11 mEq/L.

Recent insights suggest even sodium levels below 135 mEq/L (the lower limit of normal) carry prognostic significance. Levels measured below 135 mEq/L are evident in up to 50% of patients with ascites [18]. In the literature, the prevalence of hyponatremia in DCLD patients is described as being around 70-73% [11, 14]. However, Angeli et al. found the prevalence of hyponatremia was 21.6% among patients with cirrhosis and ascites [19]. Kim et al. discovered the prevalence of dilutional hyponatremia, defined by serum sodium concentrations below 135 mmol/L, 130 mmol/L, and 125 mmol/L, stood at 20.8%, 14.9%, and 12.2%, respectively [15]. In contrast, this study found that, among patients who had had DCLD for less than 10 years, 35 individuals (36.8%) had hyponatremia, whereas, in patients who had suffered from DCLD for 10 years or more, 13 individuals (34.2%) displayed hyponatremia.

In one Nepali study, 47 of the 114 patients under assessment for chronic liver disease exhibited hyponatremia, constituting 41.22% of the cohort, which had an average age of 53.44±7.57 years [20]. In another study, conducted by Amna et al. in Pakistan, the prevalence of hyponatremia in the study group (36.9%) and distribution of hyponatremia severity was as follows: 9.2% mild, 21.5% moderate, and 6.2% severe [21].

The severity of hyponatremia in cirrhosis positively correlates with rates of both morbidity and mortality. The current standard therapy for hyponatremia, which limits fluid intake to 1.5 L per day, is rarely successful. Alternative strategies, including administration of albumin and vaptans, which operate by selectively counteracting the effects of arginine vasopressin on vasopressin-2 receptors in kidney tubules, have been assessed for effectiveness in treating hyponatremia [22,23]. Vaptan usage in patients with advanced liver disease is constrained by vaptan’s hepatotoxic effects; however, brief treatments with vaptan correlate with a noticeable increase in renal solute-free water excretion and improvement of hyponatremia [24-26].

Although the study's results are interesting, it had several limitations. First, this study was specifically conducted within the Pakistani population, so our results may not be generalizable to other settings, and therefore further investigations should be carried out to establish generalizability. Furthermore, this observational study was restricted to a single center in South Asia. To enhance the robustness of current findings and promote external validity, this study strongly recommends further large-scale, multi-center studies.

## Conclusions

The prevalence of hyponatremia was notably elevated among participants in this study who suffered from DCLD. Age and BMI were the most common risk factors for hyponatremia in patients with DCLD. This study thus recommends patients with DCLD should have their serum sodium levels screened at regular intervals to prevent complications, including encephalopathy in DCLD patients, particularly in younger and underweight patients.

## Appendices

Prevalence of hyponatremia in patients with decompensated chronic liver diseases (DCLD) presenting in a tertiary care hospital

Case No.          Registration No.                                   Date                            

Name:                                                             

Age:                                                                

Gender: Male            Female

BMI:                                       

Duration of DCLD:                                                     

Laboratory findings:

Sodium level:                                       mmol/L

Hyponatremia:             Yes                        No       
